# Genotypic Diversity and Virulence Traits of *Streptococcus mutans* Isolated from Carious Dentin after Partial Caries Removal and Sealing

**DOI:** 10.1155/2014/165201

**Published:** 2014-01-21

**Authors:** Nailê Damé-Teixeira, Rodrigo Alex Arthur, Clarissa Cavalcanti Fatturi Parolo, Marisa Maltz

**Affiliations:** Department of Social and Preventive Dentistry, Faculty of Dentistry, Federal University of Rio Grande do Sul, Ramiro Barcelos 2492, 90035-003 Porto Alegre, RS, Brazil

## Abstract

The aim of this study was to compare the genotypic diversity and virulence traits of *Streptococcus mutans* isolated from carious dentin before and after partial dentin caries removal (PDR) and sealing. Carious dentin samples were obtained three months before and after the PDR and cavity sealing. Up to seven isolates of each morphological type of *S. mutans* were selected and strain identity was confirmed using gtfB primer. Genotyping was performed by arbitrary primer-PCR (AP-PCR). Acidogenesis and acidurance of the genotypes were evaluated as virulence traits. A paired *t*-test and a Wilcoxon test were used to compare the virulence of genotypes. A total of 48 representative *S. mutans* isolates were genotyped (31 before and 17 after the sealing). At least one of the genotypes found before the sealing was also found on dentin after the sealing. The number of genotypes found before the sealing ranged from 2 to 3 and after the sealing from 1 to 2 genotypes. No difference was observed in the acidogenesis and acidurance between genotypes isolated before and after the sealing. In conclusion, genotypic diversity of *S. mutans* decreased after the PDR and sealing, but the virulence traits of *S. mutans* remained unchangeable.

## 1. Introduction

Stepwise excavation has been an alternative treatment for deep caries lesions since the conventional treatment based on complete dentin caries removal could generate pulp exposure and poor dental prognosis [1]. The stepwise excavation involves the partial removal of the decayed tissue, temporary sealing, reopening of the cavity, and the complete removal of the carious tissue followed by restoration [1–3]. After the sealing period, the filling is removed and complete caries removal is performed [2, 4, 5]. Partial carious dentin removal (PDR) in one session, keeping a layer of carious dentin beneath restoration, has been proposed as an alternative approach to the stepwise excavation, avoiding failures due to the loss of temporary filling, additional costs, discomfort to the patient, and the possibility of pulp exposure during the second reopening procedure [6, 7]. This treatment has shown success in clinical studies over time without the need of cavity reentry [6–8]. However, the persistence of viable bacteria in dentin after PDR has raised doubts regarding the long-term effectiveness of this treatment [9].

After the sealing period, a limited supply of nutrients is left for the bacteria that survive underneath the restoration. Strains that are fit for physical, chemical, biological, and environmental changes might dominate and get established after the sealing. Paddick et al. [10] showed that only those bacteria capable of producing the enzymes required for the cleavage of the terminal sugars from the glycoprotein were recovered from the dentin after the cavity sealing. The low nutrient supply underneath the restorations could lead to a modification of the residual biofilm.

Although a limited number of microorganisms persist under restorations a few months after the PDR and sealing [2, 3, 11, 12], some cariogenic bacteria may be found within the remaining microorganisms, such as mutans streptococci, which is currently found on sealed carious dentin [10–17]. These bacteria are capable of producing acids due to fermentation of dietary carbohydrates (acidogenesis) and surviving in that low-pH environment (acidurance), which makes them an important cariogenic microorganism related to caries initiation and progression [18]. However, the relationship between the residual *S. mutans* found on carious dentin beneath restoration after the PDR and caries progression is still unclear. Thus, it is important to better understand how the environmental changes induced by the dentin sealing affect the diversity and virulence traits of the remaining mutans streptococci.

In this context, genotyping methods based on arbitrarily primed PCR (AP-PCR) have revealed that the community of *S. mutans* isolated from saliva and dental plaque is diverse [19, 20]. Additionally, it has been shown that distinct *S. mutans* genotypes may exhibit distinct cariogenic potential [21, 22]. Therefore, the aim of this study was to compare the genotypic diversity of *S. mutans* isolated from carious dentin before and after the PDR and sealing. We hypothesize that *S. mutans* genotypes are selectively found after the PDR and sealing and exhibit low cariogenic potential.

## 2. Methods

### 2.1. Origin of the Samples

The samples were derived from a previous clinical trial. Briefly, patients (*n* = 18) with permanent molars with carious lesions located in the middle third of the dentin were selected [23]. The patients were submitted to the PDR and sealing with a biocompatible material for 3 months. Dentin samples were obtained after the PDR (before and after the sealing), by a sterile bur, transferred to and diluted in reduced transport fluid, and plated on Mitis Salivarius Bacitracin (MSB) agar (Difco Laboratories, Detroit, MI, USA). Up to 7 isolates of each morphological type found in these cultures were selected and analyzed based on colony morphology. After subculture, each isolate was stored in Brain-Heart Infusion (BHI) (HiMedia, Mumbai, India) with 15% (v/v) glycerol at −20°C for further analysis. From 18 patients, only 4 patients presented *S. mutans* isolates before and after the sealing.

### 2.2. Extraction of Genomic DNA

*Streptococcus mutans* were grown from frozen stocks on BHI agar and incubated for 24 h at 37°C in microaerophilic conditions. The genomic DNA was extracted from colonies resuspending them in 50 *μ*L of sterile ultrapure water [24]. PCR with species-specific primers to gtfB (5′-ACTACACTTTCGGGTGGCTTGG-3′ and 5′-CAGTATAAGCGCCAGTTTCATC-3′) was performed to confirm the identity of *S. mutans* isolates [19] (Invitrogen, SG, Milanese, Italy). The PCR amplifications were performed with 50 *μ*L total volume, including 1 *μ*L of the target DNA, 0.25 *μ*L of Taq DNA polymerase (5 U/*μ*L), 5 *μ*L of 10 × PCR buffer, 2.5 *μ*L of 50 mM MgCl_2_, 1 *μ*L of deoxynucleoside triphosphate mix (10 mM), and 1 *μ*L of each primer (10 mM). The amplifications were performed under the following conditions: 30 cycles of 95°C for 30 s, 59°C for 30 s, and 72°C for 1 min, followed by 1 cycle of 95°C for 30 s, 59°C for 30 s, and a final extension of 72°C for 5 min. Genomic DNA of *S. mutans* strain UA159 (provided by FIOCRUZ, Rio de Janeiro, Brazil) and ultrapure water were applied in all the PCR baths, as positive and negative controls. The PCR products were analyzed by electrophoresis on 2% agarose gel and stained with SYBR Green 1.6%, at 100 V for 45 min. Bands were visualized under UV illumination. All the chemicals were provided by Invitrogen (SG, Milanese, Italy).

### 2.3. Genotypic Analysis of *S. mutans* Isolates by AP-PCR

AP-PCR assays were performed with the arbitrary primer OPA 02 (5′-TGCCGAGCTG-3′) [19]. The DNA amplification occurred under the following conditions: 95°C for 2 min, for initial denaturation, and 45 cycles of 94°C for 30 s, 36°C for 30 s, and 72°C for 1 min, with a final extension at 72°C for 5 min. *Streptococcus mutans* strain UA159 and ultrapure water were used as controls. Products of AP-PCR were analyzed by electrophoresis on 1% agarose gel and stained with SYBR Green 1.6%, at 96 V for 4 hours.

### 2.4. Virulence Factors Analysis

The virulence traits of genotypes were evaluated as described elsewhere [19]. Frozen stocks of each *S. mutans* genotype were regrown on BHI agar plates and incubated at 37°C for 24 h. Two loops of 1 *μ*L were inoculated into 30 mL of BHI broth supplemented with 1% glucose and incubated at 37°C for 18 h. In order to evaluate the ability of *S. mutans* genotypes to lower the suspension pH through glycolysis, 10 mL of the microbial suspension was centrifuged and resuspended in 50 mM KCl/1 mM MgCl_2_ (Synth, São Paulo, Brazil). The pH of the solution was adjusted to around 7.0, and glucose was added to a final concentration of 55.5 mM. The pH was monitored for 180 min using a glass electrode previously calibrated with pH standards (pH 4.0 and 6.8). The area above the curve (AAC) was determined considering pH 6.5 as a cutoff point. The experiment was performed in duplicate and *S. mutans* UA 159 was used as a control in all the tests.

The ability of *S. mutans* genotypes to tolerate acidic environments was evaluated using 10 mL of the overnight growth suspension (described above) for 18 h in BHI broth/glucose. This suspension was diluted to 1 : 20 in BHI/glucose, and the growth was monitored until OD_550_ = 0.5. The suspensions were then centrifuged, and the pellets were washed once with 0.1 M glycine buffer (pH 7.0) (Nuclear, São Paulo, Brazil). The washed pellets were then resuspended in 0.1 M glycine buffer pH 2.8, 5.0, and 7.0. Immediately after the resuspension (time zero) and after 30 min and 60 min of incubation at 37°C, aliquots were serially diluted in phosphate buffer (pH 7.2), plated on BHI agar, and incubated at 37°C for 48 h. Cell viability at each time point was expressed as the percent of growth in relation to time zero.

### 2.5. Data Analysis

Images of AP-PCR fingerprints were captured by a digital camera (Canon Inc., Tokyo, Japan) and stored in Image File Format for visual analysis. For analysis of the *S. mutans* genotypic profiles from the same patient, AP-PCR products from the isolates obtained before and after the sealing were always resolved side by side in the same gel for visual comparisons. Thus, genotypic diversity was compared among isolates before and after the sealing samples. Isolates were considered as having the same genotypic identity when they presented identical AP-PCR product-size profiles. Later, a side-by-side analysis on the same gel was performed using each genotype (duplicate) from all the patients to verify the similarities between *S. mutans* isolates from different patients. Two blinded and calibrated examiners performed the visual analysis. Cohen's Kappa value was 0.76. Double cases were discussed and a consensus was reached. Genotypes (number and proportion) were described in each patient before and after the sealing (descriptive analysis). Data were transformed to log_10_ due to data dispersion, and the normal distribution was confirmed using a histogram and Kolmogorov-Smirnov test, except for the pH 2.8 analysis. A paired *t*-test and a Wilcoxon test were used to compare the virulence of genotypes (acidogenesis and acidurance) found before and after the sealing. The significance level was set at 5%. The virulence-traits data were analyzed by (1) the paired comparison between genotypes found before the sealing with the same genotypes found after the sealing (genotypes A, D, E, G, and I) and by (2) the comparison between the means of acidogenesis and acidurance of all the genotypes found before the sealing with all the genotypes found after the sealing. Statistical tests were performed in SPSS 18.0 for Windows (IBM SPSS Statistics).

### 2.6. Ethical Considerations

The protocol of the clinical trial was approved by the ethics committee of the Faculty of Dentistry from the Federal University of Rio Grande do Sul (process no. 19218). Informed and written consent was obtained from all the individuals. All the participants received the treatment for basic dental needs.

## 3. Results

**Figure 1:**
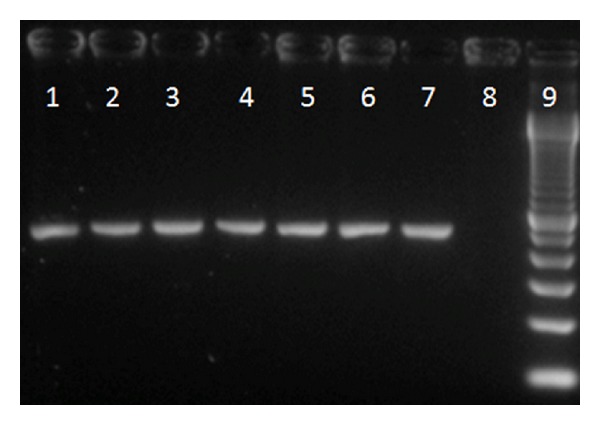
*S. mutans* identification by species-specific gtfB primer. Lanes 1 to 6: isolates of *S. mutans*; lanes 7 and 8 correspond to the positive control (*S. mutans* UA 159) and negative control (water), respectively; lane 9: 100-bp DNA ladder.

**Figure 2:**
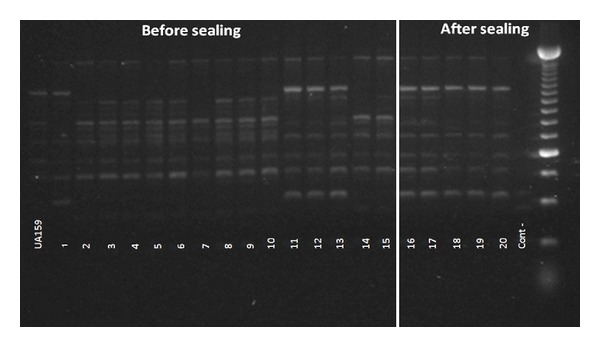
Electrophoresis gel image from AP-PCR-generated fingerprints using primer OPA-02 of *S. mutans* reference strain (UA159) and isolates from carious dentin before and after sealing. Lanes 1 to 15 correspond to the *S. mutans* isolates before sealing; lanes 16 to 20 correspond to the *S. mutans* isolates after sealing; lane 21: negative control (water); lane 22: 250-bp DNA ladder.

A total of 48 *S. mutans* isolates were obtained from carious dentin corresponding to 31 found before and 17 found after the sealing (Table 1). All of them were identified as being *S. mutans* by species-specific PCR (Figure 1). The molecular weight fragments generated by AP-PCR from the studied patients range between 490 and 5000 bp generating 9–12 DNA bands. The genotypic diversity of *S. mutans* found in one patient (patient 1) is shown in Figure 2. The number of genotypes found per patient varies between 2-3 and 1-2 before and after the sealing, respectively. At least one of the genotypes found before the sealing was also found on dentin after the sealing in all the patients. Only one patient presented one new genotype after the sealing (patient 3). Two patients (1 and 4) showed reduction in the number of genotypes before and after the sealing. In another patient (patient 2), the same genotypes were found before and after the sealing, although their proportion was different, with predominance of genotype D (Table 1). For patient 3, only one of the genotypes identified before the sealing was found after the sealing. That genotype was the most prevalent, and it colonized the dentin in addition to a new genotype exclusively found after the sealing (Table 1). No similarities were found between *S. mutans* isolates from different patients.

The results of the analysis of the virulence traits are described in Table 2 (acidogenesis) and Table 3 (acidurance). In the acidogenesis analysis, no difference was observed in the AAC neither when the comparisons were made considering genotypes found before and after the sealing within the same patient (*P* = 0.2) nor when the comparisons were made considering all genotypes found before and after the sealing (*P* = 0.08). Regarding the acidurance, no difference was observed in the growth of the genotypes at different pH when the analysis was performed considering paired genotypes found before and after the sealing.

## 4. Discussion

This study aimed to compare the genotypic diversity and the virulence traits of *S. mutans* in carious dentin after PDR before and after the sealing, using AP-PCR fingerprinting analysis. We observed a reduction in the number of *S. mutans* isolates found after the sealing (Table 1). This is consistent with a reduction in counts of viable cells on carious dentin found after the cavity sealing [23]. AP-PCR has been used to evaluate the genotypic profile of *S. mutans* from saliva, biofilm, tongue, and dentin [19–21, 25–30]. The validity of the AP-PCR technique in the genotypic identification of microorganisms is assured by several comparisons made with other genotyping techniques [20, 31, 32].

Previous studies have shown a decrease in bacterial diversity [10, 33] after the PDR and sealing. A shift in the bacterial genotypes has also been observed [10], but no studies evaluating the genotypic diversity of *S. mutans* were found. In the present study, an altered genotypic diversity was observed after sealing the carious dentin.

It has been discussed that the residual bacteria found after the sealing have limited access to external nutrients. They are fed mainly by glycoproteins provided by the pulp and that nutrient restriction exerts a selective pressure over the residual bacteria [10]. In that scenario, a metabolism shift has been found in bacteria isolated from carious dentin after the sealing [10]. It is also likely that components of dead bacteria, which did not survive after the sealing, may contribute as the source of nutrients to those bacteria that survived below the restorations [10]. Considering that our dentin samples were collected 3 months after the sealing, these findings might explain the altered genotype prevalence found within different patients after the dentin sealing (Table 1). Some genotypes (B and F) were the most prevalent in dentin before the sealing; however, they were not detected after the dentin sealing (Table 1). The undetected levels of these genotypes after the sealing might have enhanced the competitiveness of other genotypes (A and G) that were most prevalent after the sealing. In patient 3 (Table 1), there was a shift in the prevalence of genotypes found before and after the sealing. We believe that the reduction in the proportion of genotypes D and J has created a better condition for the growth of genotypes E and I. Interestingly, only genotype H was found on dentin samples after the sealing. It means that the genotype was below the detection limit of the microbiological method used [34] on dentin samples before the sealing. Moreover, some rare or transient genotypes in a single sample might be missed in a complex microbiota, which could explain the appearance of a new genotype only after the sealing [35]. The sealing of the cavity reduced the prevalence of genotype F, which might have created better conditions for the growth of genotypes G and H. In an open environment, such as a dental caries cavity, the microbiota of the biofilm above the dentin is continuously exposed to the dietary carbohydrate that provides nutrients for the growth of the diverse oral flora. However, the sealing of carious cavity was responsible for the selection of genotypes that were capable of surviving in the presence of low nutrient availability. Besides reduced availability of nutrients during the sealing, the relative simplicity and homogeneity of these nutrients significantly affect the microbiota surviving under the restorations [10, 33].

Additionally, microbiological studies have reported a significant decrease in the bacterial infection in the residual carious dentin after the cavity sealing [2, 3, 11, 12]. However, despite this microbial residual contamination found after the sealing, the carious dentin becomes harder and drier, both characteristics of inactive lesions [2]. Therefore, the reduction in the number of *S. mutans* genotypes found after the cavity sealing represents to some extent the low number of genotypes frequently found in caries-free individuals [25, 27, 28].

It remains unclear, though, whether the bacteria underneath restorations represent some danger to the longevity of restorations and if they are more virulent than the bacteria prior to the sealing. In this study, no statistical difference was found regarding the virulence traits of *S. mutans* isolated before and after the sealing. Even under a low-nutrient-availability condition, the role of specific phenotypic traits on the prevalence of these genotypes remains the same. That means those genotypes found after the sealing might be metabolically active if a source of external carbohydrates is provided, and, in addition to that, those genotypes are still capable of developing an acid-tolerance response to the acidic condition. Thus, it is important to point out the need for a perfectly sealed restoration.

Moreover, acidurance and acidogenesis of genotypes found before and after the sealing (A, D, E, G, and I) were not different which means that equal genotype presented equal phenotype. In contrast, several studies have shown that distinct *S. mutans* genotypes might show distinct acidurance [21, 22]. The results of the acidurance analysis in the present study suggest that the higher pH observed in sealed carious dentin [36] is due to the decrease in the number of these acidogenic bacteria and the limited access to nutrients [10] and not by their lower capacity of acid production.

Therefore, it is important to know if the biofilm that survived underneath the restorations remains potentially cariogenic [10, 37]. According to Takahashi and Nyvad [38], it is not only important to describe which bacteria are involved in caries, but it is also important to know what is their function [38]. Although *S. mutans* is one important cariogenic microorganism related to caries, only 4 out of 18 patients showed this microorganism after the sealing. Besides this, the *S. mutans* that survived presents similar virulence in comparison to the initial *S. mutans*.

In conclusion, genotypic diversity of *S. mutans* was reduced after PDR and sealing using AP-PCR fingerprinting analysis. Additionally, there was not any difference in acidurance and acidogenesis between genotypes found before and after the sealing. Genotypes found after the PDR and sealing have the same cariogenic potential of those found before the sealing.

## Figures and Tables

**Table 1:** Number of isolates and genotypes (%) of *S. mutans *isolated per patient from carious dentin before and after sealing.

Patients	Before sealing		After sealing	
	Number of isolates	Genotypes (%)	Number of isolates	Genotypes (%)
1	15	A (26.7) B (60.0) C (13.3)	5	A (100.0) — —
2	11	D (54.5) E (45.4)	3	D (66.6) E (33.3)
3	3	F (66.6) G (33.3) —	8	— G (87.5) H (12.5)
4	2	I (50.0) J (50.0)	1	I (100.0) —

Total	31	9	17	6

Distinct letters show different genotypes in each patient. The designation of genotypes by letters (A, B, C, etc.) is only valid within each patient.

**Table 2:** Acidogenesis (AAC; pH 6.5 as cutoff point) of *S. mutans *genotypes isolated from carious dentin before and after sealing.

	Before sealing Mean (SD)	After sealing Mean (SD)	*P*
Paired genotypes	87.86 (10.31) (*n* = 5)	93.75 (9.34) (*n* = 5)	0.2
All genotypes	86.25 (3.40) (*n* = 9)	95.01 (4.34) (*n* = 6)	0.08

AAC: area above the curve.

**Table 3:** Acidurance of *S. mutans* genotypes isolated from carious dentin before and after sealing.

Time/pH	Paired genotypes			All genotypes		
	Before sealing (*n* = 5)	After sealing (*n* = 5)	*P*	Before sealing (*n* = 9)	After sealing (*n* = 6)	*P*
	% grown (min–max)	% grown (min–max)		% grown (min–max)	% grown (min–max)	
30 min						
*pH 7.2	96.1 (82.9–103.0)	91.5 (86.5–97.8)	0.3	95.4 (83.5–100.4)	92.2 (86.5–95.7)	0.5
*pH 5.0	85.3 (35.6–100.9)	98.2 (93.9–101.8)	0.3	91.1 (62.6–102.2)	96.5 (91.9–101.1)	0.6
^†^pH 2.8	63.4 (35.6–145.7)	38.2 (10.1–51.7)	0.1	48.7 (38.8–72.7)	34.6 (10.1–47.0)	0.3

60 min						
*pH 7.2	89.7 (64.9–103.9)	93.4 (90.1–97.4)	0.6	93.3 (83.5–102.6)	94.2 (90.6–96.9)	0.9
*pH 5.0	90.1 (62.4–105.2)	77.1 (45.5–98.5)	0.4	80.8 (52.9–99.1)	84.5 (71.1–98.5)	0.8
^†^pH 2.8	35.6 (00.0–93.3)	22.6 (00.0–58.3)	0.5	36.7 (19.3–67.8)	20.3 (00.0–44.9)	0.3

Percent of bacterial growth in relation to time zero (100%) in pH 7.2, pH 5.0, and pH 2.8.

*Paired *t*-test.

^†^Wilcoxon nonparametric test.
